# Pathological and Molecular Features of Glioblastoma and Its Peritumoral Tissue

**DOI:** 10.3390/cancers11040469

**Published:** 2019-04-03

**Authors:** Alessio D’Alessio, Gabriella Proietti, Gigliola Sica, Bianca Maria Scicchitano

**Affiliations:** Istituto di Istologia ed Embriologia, Università Cattolica del Sacro Cuore, Fondazione Policlinico Universitario “Agostino Gemelli”, IRCCS, 00168 Roma, Italy; gabriella.proietti@unicatt.it (G.P.); gigliola.sica@unicatt.it (G.S.); biancamaria.scicchitano@unicatt.it (M.S.)

**Keywords:** biomarkers, chemotherapy, microRNA, cancer stem cells, central nervous system, glioma, GBM, peritumoral tissue

## Abstract

Glioblastoma (GBM) is one of the most aggressive and lethal human brain tumors. At present, GBMs are divided in primary and secondary on the basis of the mutational status of the isocitrate dehydrogenase (*IDH*) genes. In addition, *IDH1* and *IDH2* mutations are considered crucial to better define the prognosis. Although primary and secondary GBMs are histologically indistinguishable, they retain distinct genetic alterations that account for different evolution of the tumor. The high invasiveness, the propensity to disperse throughout the brain parenchyma, and the elevated vascularity make these tumors extremely recidivist, resulting in a short patient median survival even after surgical resection and chemoradiotherapy. Furthermore, GBM is considered an immunologically cold tumor. Several studies highlight a highly immunosuppressive tumor microenvironment that promotes recurrence and poor prognosis. Deeper insight into the tumor immune microenvironment, together with the recent discovery of a conventional lymphatic system in the central nervous system (CNS), led to new immunotherapeutic strategies. In the last two decades, experimental evidence from different groups proved the existence of cancer stem cells (CSCs), also known as tumor-initiating cells, that may play an active role in tumor development and progression. Recent findings also indicated the presence of highly infiltrative CSCs in the peritumoral region of GBM. This region appears to play a key role in tumor growing and recurrence. However, until recently, few studies investigated the biomolecular characteristics of the peritumoral tissue. The aim of this review is to recapitulate the pathological features of GBM and of the peritumoral region associated with progression and recurrence.

## 1. Introduction

GBM is the most common malignant primary brain cancer [1,2]. Despite the growing experimental investigation in this field and the improved therapeutic strategies, GBM remains essentially incurable, with an overall survival time ranging from 12 to 18 months [3], as less than 5% of patients survive longer than five years after diagnosis [4,5]. The poor prognosis of GBM and its high frequency of recurrences forced researchers to pursue novel fields of investigation in the area of molecular biology for hindering this disease. Nevertheless, the majority of studies over the years focused on the core tumor area of GBM, whereas less is known about the peritumoral area that may also be infiltrated by tumor cells. Recent studies focused on the characterization of this, at first glance, “normal tissue” surrounding GBM to better define its role in GBM progression and search for potential therapeutic targets [6,7,8,9,10,11,12,13,14,15]. Moreover, deeper investigations on the tumor immune microenvironment, together with the recent discovery in the meninges of a central nervous system (CNS) conventional lymphatic system, provided a new impetus to immunotherapeutic strategies, which emerged as promising targeted and less toxic treatments [16]. This work aims at reviewing recent findings on both the morphological and molecular characterization of GBM and its surrounding tissue, including the presence and the role played by cancer stem cells (CSCs).

## 2. Pathological and Molecular Features of GBM

Gliomas include a variety of primary malignant tumors of the CNS that develop either from glial cells, such as astrocytes, oligodendrocytes, microglia, and ependymal cells, or from a subpopulation of CSCs residing in the tissue. Among the different malignant gliomas, GBM, which accounts for about 60–70% of all gliomas, is classified as a World Health Organization (WHO) grade IV tumor based on histopathological features, and it represents the most frequent and malignant tumor of the CNS, affecting both children and adults with a slight predominance in males [17]. GBM is defined as a diffuse glioma, characterized by a high aptitude to infiltrate the surrounding brain tissue. In addition, molecular profile of GBM has been used to improve classification [18]. In particular, different clinically relevant GBM subtypes (proneural, neural, classical, and mesenchymal) that were identified on the basis of the gene expression profiles are essential to develop specific clinical strategies [19]. According to recent discoveries, GBMs are now subdivided based on the mutational state of isocitrate dehydrogenase (*IDH*) genes in *IDH* wild type which corresponds most frequently with the clinically defined primary or de novo GBM, *IDH* mutant which corresponds to the so-called secondary GBM, and those not otherwise specified (NOS) for which the *IDH* status could not be determined [17]. Primary and secondary GBMs show similar histological characteristics but they differ in genetic and epigenetic profiles and are thought to develop from different cells of origin. They have a significantly different clinical outcome; in fact, tumors with mutated *IDH1* and *IDH2* have improved prognosis [20]. In order to diagnose GBM, patients are usually subjected to a preliminary neurological exam to identify which area of the brain may be affected by the tumor. This is commonly followed by imaging tests, such as computed tomography (CT) and magnetic resonance imaging (MRI), to determine the location and the size of the tumor. Finally, the histopathological analysis performed on a tissue sample will ascertain the type of tumor and its aggressiveness. The primary treatment of GBM-affected patients is undergoing surgical resection followed by radio and temozolomide (TMZ)-based therapy [4]. However, the extreme heterogeneity of these tumors makes cancer therapies increasingly challenging. Together with inter-tumor heterogeneity, intra-tumor heterogeneity represents a crucial field of investigation of GBM since it requires the study and the comprehension of an assortment of biomolecular features such as genetic and epigenetic abnormalities, the identification of precise molecular markers, and the rate of cell growth and death of tumor cells [21,22,23]. Histological features of GBM include marked hypercellularity, nuclear atypia, microvascular proliferation, and necrosis. The tumor shows palisading of tumor cells around necrotic foci; in addition, GBM harbors CSCs. Although the histological analysis remains essential in the diagnosis of gliomas, recent discoveries especially in the field of genetics strongly improved our understanding of these tumors. In addition to the mutational status of *IDH1/2* and enzymes involved in a variety of metabolic processes, such as the production of redox species and epigenetic mechanisms, and DNA repair [24,25,26,27], the 6-*O*-methylguanine DNA methyltransferase (MGMT), involved in DNA repair, is another key predictive and prognostic marker for the treatment of GBM [28]. GBM patients with promoter methylation of this gene, who are treated with alkylating agents, show longer survival compared to patients in which the MGMT promoter was not methylated [29]. Among the different genetic alterations found in GBM, those targeting the transmembrane epidermal growth factor receptors (EGFRs) play a crucial role. In fact, approximately 40% of tumors show EGFR amplification and may express a truncated form of receptor due to genomic deletions. Interestingly, these alterations highly correlate with patient survival and response to treatment [30,31]. In addition, EGFR alterations are concurrent with amplification and/or mutations of platelet-derived growth factor receptor A [30]. Understanding the biological mechanisms occurring in GBM is fundamental for clarifying processes involved in carcinogenesis and progression of the tumor, as well as for developing clinical strategies aimed to target cancer cells.

## 3. The Immune Microenvironmental Landscape in GBM

The immune system patrols and monitors the body in order to defend it from tumors in a process known as cancer immunosurveillance [32,33]. Notably, this process occurs also in the CNS [34,35], in apparent conflict with its traditional view of an immune-privileged site. In fact, the brain was always considered a low immune responsive organ [36,37], due to the presence of a highly selective physical blood–brain barrier (BBB), made up by endothelial cells (ECs) stitched together by tight junctions, their basement membrane, surrounding pericytes, and astroglial endfeet processes [38], as well as low major histocompatibility complex (MHC) class I and II expression [39] and the apparent absence of a conventional lymphatic system [40]. Recently, the dogma of the brain immune privilege and tolerance was debunked by the discovery in the meninges of a CNS conventional lymphatic system [16]. Intra and extra cranial lymphatic vessels drain brain tissue fluid and transport it into the bloodstream, through arachnoid granulations located along the superior sagittal and the transverse sinuses, or crossing the cribriform plate, in the nasal lymphatic vasculature and then into deep cervical lymph nodes. The macromolecules and the immune cells from cerebrospinal fluid and brain parenchyma reach the deep cervical lymph nodes, even if their exact route is still unclear, thereby engaging the peripheral immune system [41,42]. Moreover, pathological stimuli, such as tumor growth, induce changes in the BBB, which physiologically confers to CNS blood vessels a selective permeability, opening the door for several types of immune cells. These acquisitions justify the recruitment of immune infiltrate of T lymphocytes, dendritic cells, natural killer (NK) cells, and microglia/macrophages in brain tumors [43] and their potential to elicit tumor-specific immune responses [44]. Specifically, GBM displays a complex relationship between immune surveillance, tumor-induced immunosuppression, and cancer development. In spite of the presence of an immune infiltrate, a highly immunosuppressive tumor microenvironment is present in GBM, fostering recurrence and poor prognosis [45,46,47]. The tumor microenvironment is the environment that encircles cancer cells. This consists of stromal, vascular, and immune cells, together with secreted factors and the extracellular matrix. The immune infiltrate is mainly constituted by lymphocytes, macrophages, and microglia. In particular, macrophages and microglia represent 30–50% of the tumor mass, and their phenotypes and functions display deep modifications induced by tumor cells [48]. At present, several clinical trials are ongoing in which GBM patients’ immune systems are stimulated to kill tumor cells using, for example, dendritic cell vaccines (https://clinicaltrials.gov/).

### 3.1. GBM-Associated Microglia and Macrophages 

Brain macrophages are considered the resident immune cells of the CNS, involved in brain homeostasis and immune responses [49]. This group includes microglia, perivascular macrophages, meningeal macrophages, macrophages of the circumventricular organs, and macrophages of the choroid plexus. In GBM, microglial cells and infiltrating macrophages accumulate within and around the tumor mass, but they are ineffective in fighting tumor development or can even bolster it. The GBM-associated microglia and macrophages (GAMMs) system is composed of cluster of differentiation (CD)11b^+^/CD45^dim^ activated resident microglia (15%), mainly localized in peritumoral areas, and by an infiltrate of CD11b^+^/CD45^high^ peripheral monocyte-derived macrophages (85%), located in perivascular regions [50]. GAMM recruitment is mediated by many chemoattractants, such as the monocyte chemoattractant protein 1, the glial cell-derived neurotrophic factor, the granulocyte macrophage colony-stimulating factor [48], different molecules present in the secretome such as the hepatocyte growth factor/scatter factor [51], and the integrin ligands osteopontin and lactadherin [52]. GAMMs present considerable diversity and plasticity, and display a partly understood unique phenotype, only partially ascribable to inflammatory (M1) or alternative (M2) polarization expression patterns [53]. In fact, GAMMs show typical hints of an alternative macrophage activation, as they can inhibit inflammation via transforming growth factor (TGF) β1, arginase 1 (ARG1), and interleukin 10 (IL-10) production and shape the tumor microenvironment through secretion of vascular endothelial growth factor (VEGF) and matrix metalloproteases (MP); meanwhile, they show classical macrophage activation aspects, such as the production of pro-inflammatory molecules (IL-1β, tumor necrosis factor, IL-6, and IL-12), along with the induction of T helper 1 (Th1)-mediated immune responses [54,55,56,57,58]. To date, it is well known that the abundance of GAMMs positively correlates with GBM invasiveness, immunosuppression, and patients’ poor prognosis [59,60], making these cells a good target for immunotherapeutic strategies. 

### 3.2. Myeloid-Derived Suppressor Cells

Myeloid-derived suppressor cells (MDSCs) are a heterogeneous group of cells defined by their myeloid lineage, immature state, and ability to potently suppress T-cell responses [61]. In many solid tumors, including gliomas, CD11b^+^CD14^+^CD33^+^HLA-DR^−^/^low^Co-receptor^−^/^low^ monocyte-MDSCs (M-MDSC), CD11b^+^CD15^+^CD33^+^Lin^−^HLA-DR^−^/^low^ polymononuclear-MDSCs (PMN-MDSC), and a more immature subset of CD3^−^CD14^−^CD15^−^CD19 ^−^CD56^−^CD33^+^CD11b^+^ early-MDSCs (e-MDSC) were described [45,62]. MDSCs contribute to tumor immune evasion in different ways. They suppress first-line defense, for example, inhibiting the NK cell activation receptor NKG2D and preventing IFNγ production by NK cells, in the presence of TGF-β [63]. MDSCs dwindle adaptive immune responses via the induction of FOXP3^+^ regulatory T cells (Treg), by the polarization of T cells toward a tumor-promoting type 2 phenotype, by the inhibition of T-cell function and proliferation through production of ARG1 and inducible nitric oxide (NO) synthase 2 (iNOS2) or, in an l-arginine-independent manner, via reactive oxygen species (ROS) and TGF-β production, cysteine depletion, and L-selectin (CD62L) downregulation [64,65]. Finally, MDCSs promote tumor growth favoring angiogenesis and vasculogenesis and positively correlate with poor outcomes in patients with solid tumors [66]. In GBM, elevated levels of PMN-MDCSs were detected in tumor tissue and blood, showing high expression of S100A8/9 and arginase that correlate with T function suppression [67]. Both MDSCs in peripheral blood and those at the tumor site play a major role in GBM-induced T-cell suppression. It was recently shown that MDSCs within brain tumors undergo transmembrane protein programmed death ligand 1 (PD-L1) upregulation, while tumor-derived CD4^+^ T cells express high levels of PD-1. The PD-1/PD-L1 interaction results in T-cell exhaustion, inhibiting antitumor immune responses [68]. 

### 3.3. GBM-Infiltrating Lymphocytes

GBM immune infiltrating cells include lymphocytes (tumor-infiltrating lymphocytes, TILs), the key players of adaptive cellular immune defense, particularly CD8+ T cytotoxic (Tc) and CD4+ T helper (Th). Apart from a long-term resident population of CD8^+^CD25^+^CD45RO^+^CD28^+^CD26L^+^CCR7^+^ memory T cells, CD8^+^CD3^+^ and CD4^+^CD3^+^ TILs were described in GBM, especially in fibrinogen-positive areas, where vessels are no longer watertight, and are positively associated with a longer clinical survival [69,70]. Conversely, inactivated CD8^+^CD25^−^ and CD4^+^CD25^+^FOXP3^+^ Treg cells were found within the tumor tissue. Treg cells are able to suppress the antitumor immune response and induce tolerance by inhibiting the proliferation of effector T cells and their secretion of cytotoxic cytokines; thus, they correlate with worse prognosis [71]. NK cells are another type of cytotoxic lymphocytes infiltrating GBM tissue. These large granular lymphocytes exert biological functions ascribable to both innate and adaptive immunity against viral infected and tumor cells [72]. CD3^−^CD56^+^CD16^+^ NKs are activated by recognition of stress-induced MHC I or MHC I-like proteins on the cell surface, and they induce direct cytotoxicity of target cells [73]. In GBM cells, genomic instability and metabolic derangements induce the expression of ligands of NK group 2 member D (NKG2D) receptor, called NKG2DLs, such as MHC class I-related chains A and B and the UL16-binding protein family [74]. Recognition of these ligands should trigger one of the main NK lysis mechanisms involved during the elimination phase of innate immune tumor surveillance. Some authors also reported NK cells’ capability of killing GBM cells with stem-like properties [75,76]. Actually, GBM escapes NK immune surveillance due to TGF-β-mediated downregulation of NKG2D and by shedding NKG2DLs from the cell surface through MP [77,78]. Ultimately, GBM-infiltrating NK cells are non-functional, and, in the last few years, many immunotherapeutic efforts targeted restoring and potentiating their antitumor response.

## 4. MicroRNAs in the Pathogenesis of GBM

Because of the failure of common therapies, many efforts focused on the identification of molecular targets in support of the diagnosis and the treatment of GBM. The small highly conserved non-coding microRNAs (miRNAs) raised increasing interest among scientists seeking novel therapeutic targets to neutralize GBM. MicroRNAs represent master and versatile regulators of gene expression [79], both in physiological and pathological conditions, due to their capacity to achieve post-transcriptional silencing of target genes, including tumor suppressors or oncogenes. The accessibility to the latest technologically advanced tools dramatically improved the appraisal of microRNA expression patterns in many tumors, including those affecting the brain tissue. Among over 240 miRNA molecules identified in various GBM samples, most of them are upregulated while a few are downregulated compared to normal tissue [80,81,82,83]. While precise mechanisms linking miRNAs to their biological functions are still uncertain, it is clear that the dysregulation of the expression profile of these molecules plays a crucial role in cancer development and progression [84,85]. While the mechanisms of action of some miRNAs expressed in GBM were reported in the literature, for others, the functional activities are yet to be fully characterized. As for other type of genes, some microRNAs can function as oncogenes or oncomiRs, while others show antioncogenic features. The contribution of miRNAs to the development and progression of gliomas refers to their regulation of crucial mechanisms, such as apoptosis [86], proliferation and the cell cycle [87,88], the remodeling of the extracellular matrix, tumor infiltration and angiogenesis [86,89], invasiveness [90], stem-cell renewal [91,92,93], and DNA repair [93]. Among the first microRNAs reported to be overexpressed in GBM compared to the normal brain tissue is miR-21, whose main function is to prevent the activation of the caspase-dependent apoptotic pathway [80], contributing to the onset of the malignant phenotype. Other microRNAs found upregulated in GBM, including miRNA-10b, microRNA-221, and microRNA-222, were linked to the regulation of the cell cycle and invasion of GBM cells [88,90]. Concurrently, some microRNAs were found downregulated in GBM, such as miR-181b, which was linked to GBM cell resistance to teniposide [94], and miR-125b, whose reduction favors the invasion of GBM cells by inducing MP activity [95]. For a more recent and exhaustive overview of the role of microRNAs in gliomas, see Reference [96]. These findings spotlighted miRNAs as a potential target for new therapeutic approaches in gliomas, and the investigation in this field proceeds at a swift pace.

## 5. Biomolecular Characteristics of Peritumoral Tissue

**Figure 1:**
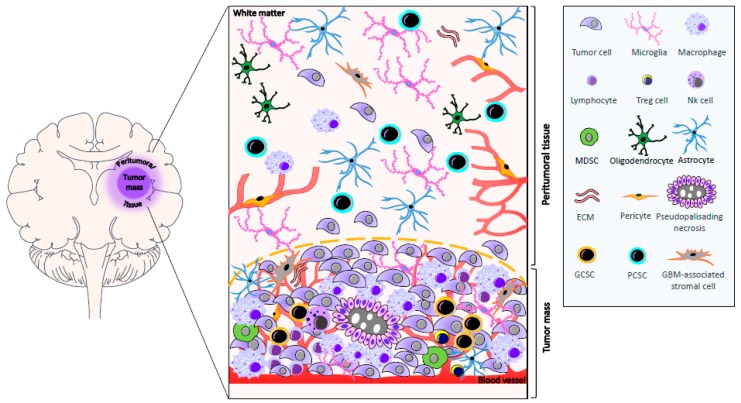
Representation of the main cell populations present in GBM and peritumoral tissue. The tumor mass is characterized by highly proliferating tumor cells, necrosis, and neoangiogenesis. Large areas of necrosis are surrounded by tumor cells arranged in a pseudopalisading pattern. Tumor microenvironment includes ECs and pericytes, reactive astrocytes, GBM-associated stromal cells, extracellular matrix, immune infiltrate of T lymphocytes and Treg cells, myeloid-derived suppressor cells (MDSCs), NK cells, activated resident microglia, peripheral monocyte-derived macrophages, and GBM cancer stem cells (GCSCs). The peritumoral tissue may present tumoral cells and it harbors ECs and pericytes, GBM-associated stromal cells, extracellular matrix, reactive astrocytes, oligodendrocytes, inflammatory cells, and peritumoral tissue cancer stem cells (PCSCs). In addition, persistent neurons are found in the white matter (not shown).

More than 90% of GBMs recur within 2–3 cm of the resection margin [97]. The area surrounding the tumor represents the invasion front of GBM into the neighboring tissue and, for this reason, it is assuming a growing interest in translational research. While, in the past years, few data were present in the literature regarding the biomolecular characterization of peritumoral tissue, recently, several studies focused on this topic with the aim of optimizing surgical resection, better defining its role in GBM progression, and finding new therapeutic targets [6,7,8,9,10,11,12,13,14,15]. Nevertheless, it is worth mentioning that, in many studies, the definition of peritumoral tissue is not always clear and unequivocal. Lemée et al. radiologically defined the peritumoral brain zone as the area surrounding GBM in the absence of contrast enhancement (T_1_) in three-dimensional MRI. In addition, this area often shows a hyperintense signal in T_2_-weighted MRI and in a fluid-attenuated inversion recovery (FLAIR) scan [11]. The brain surrounding GBM may contain neoplastic cells, and it is mainly populated by reactive astrocytes, microglia, oligodendrocytes, inflammatory cells, ECs, and pericytes, and GBM-associated stromal cells [6,7,8,11,13], which have phenotypic and functional properties similar to cancer-associated fibroblasts found in carcinomas. It also contains CSCs (see Section 6). In addition, the presence of persistent neurons was reported in the white matter, which might indicate a disorder in neuronal development or migration [98], Figure 1. This region also shows edema and vascular alterations [99,100]). Interestingly, in this compartment, various molecules are present such as receptors, amino acids, activators of transcription factors, and markers of proliferation and invasion, and the level of their expression is often similar or higher than that of the tumor tissue. Brain edema contributes to morbidity and mortality, and the identification of molecular mechanisms involved in its formation might be useful to identify novel anti-edema treatments. The integrity of tight junctions of microvessel endothelium is crucial to the maintenance of the BBB. It was shown that these junctions exhibit morphological abnormalities in GBM. Moreover, in low-grade gliomas, tumor cells are able to produce factors that induce the expression of tight junctions; however, in high-grade tumors, this capability is lost, whereby tight junction proteins are underexpressed. Finally, tumor cells secrete VEGF, which induces the phosphorylation of tight-junction proteins and the opening of these junctions. VEGF may diffuse to peritumoral tissue, and cause phosphorylation of tight-junction proteins in this area, which may worsen the edema [101]. Experimental evidence suggests that NO is involved in edema formation. NO is a potent signaling molecule that increases tumor blood flow and vascular permeability. It is also involved in neovascularization. NO is mainly produced by vascular endothelium, although glial cells may be induced to secrete it. In this regard, it was demonstrated that NO synthase (NOS) is expressed in brain tumors and in the brain tissue adjacent to the tumor, as well as being involved in edema. Nevertheless, the expression of endothelial NOS and brain NOS tends to decrease away from the tumor, suggesting that it plays a central role in NO production [102]. An important regulator of NO production in tumors is the inducible isoform of NOS (iNOS, NOS2). Inducible NOS-derived NO was linked both to tumor progression and antitumor activity. In particular, it was demonstrated that the tumorigenicity of glioma cancer stem cells (GCSs), but not of non-GCSs, depends on the expression of iNOS [103]. Adenosine is present in human glioma extracellular spaces. It is a marker of astrocyte purine metabolism, involved in the development of cancer through several mechanisms mediated by its four receptor subtypes. Adenosine can stimulate cell proliferation and also suppress the local anti-tumor immune response [104]. GBM and adjacent tissue, at the margin of tumor mass, show increased levels of A_1_ adenosine receptors [105]. In addition, increased levels of copper and zinc were found in the peritumoral region in which elevated A_1_ adenosine receptors are present, suggesting that, in this area, a complex biochemical reorganization occurs [106]. Cubillos et al. reported that the concentration of taurine, an amino acid that may have a protective effect or be involved in cell proliferation, was found to be higher in tumoral and peritumoral tissue of gliomas in comparison with extratumoral tissue. Nevertheless, in this paper a clear definition of the extension of peritumoral and extratumoral tissue is lacking [107]. Signal transducers and activators of transcription (STAT) proteins, which are activated by growth factors and cytokines, were shown to be present in the peritumoral area at the border between GBM and the non-invaded brain tissue [108]. Two studies by our group focused on the expression in peritumoral tissue of kinases involved in cell proliferation, differentiation, and motility. In particular, these studies involved patients with GBM who underwent “en-bloc” surgery. Tumor removal was achieved with resection margins including the neighboring apparently normal tissue. The adopted surgical technique [109] allowed us to obtain samples of the contrast enhancing lesion (usually designated as first area), of tissue surrounding the contrast enhancing lesion at a distance of <1 cm (second area), and of tissue localized at a distance starting from 1 cm up to 3.5 cm from the edge of GBM (third area). Extracellular signal-regulated kinases (ERKs) have a crucial role in transducing growth factor signals. Total ERK1/2 was expressed both in GBM and in peritumoral tissue. It was present in neoplastic cells, in reactive glial cells, and in apparently normal glial cells (i.e., cells that, from the histological point of view, did not show signs of transformation). The level of total ERK1/2 expression was higher in the second and in the third area with respect to GBM. Activated ERK1/2 was present in both GBM and peritumoral tissue; it was not limited to neoplastic cells and reactive astrocytes, but it was observed in apparently normal cells, even in the absence of neoplastic cells. There was no significant difference in the activated ERK1/2 expression between the contrast enhancing lesion and the areas surrounding the tumor [6]. Stress-activated/c-Jun NH_2_ terminal kinases (JNKs), which can be involved in the acquisition of transformed phenotype and are commonly thought to regulate apoptosis, were also found in both GBM and peritumoral tissue. In particular, in peritumoral tissue, activated JNK expression was independent of the presence of neoplastic cells. Nestin, a class VI intermediate filament protein, is a stem-cell marker which was found to be expressed in the majority of cells in GBM but infrequently in peritumoral tissue. Univariate analysis indicated that the ratio phosphorylated JNK/nestin in the tissue at a distance <1 cm from the tumor margin influenced the patients’ survival, having a prognostic implication [7]. In 2013, Mangiola et al., on the basis of the abovementioned surgical technique, compared the expression pattern of control (white matter) and peritumoral tissue (at least 1 cm from the macroscopic tumor border), demonstrating that up to 57 genes were differentially expressed in the peritumoral tissue versus control [9]. Interestingly, these genes were also highly expressed in GBM, suggesting that GBM and apparently normal peritumoral cells share a similar gene expression profile. Moreover, peritumoral tissue shows the upregulation of genes involved in proliferation and tumor progression, while genes known to have a role in neurogenesis and to exert an anti-oncogenic function were downregulated [9]. Finally, Lama et al. reported the expression of progenitor/stem-cell markers (GD3 and NG2) in tissue localized at a distance starting from <1 cm up to ≥1 cm from tumor border, suggesting their possible involvement in pre/pro-tumorigenic events occurring in this area [13]. These findings clearly demonstrate that the peritumoral tissue, even in the absence of neoplastic cell infiltration, shows signs of biochemical reorganization and transformation. 

### The Angiogenic Process in GBM and Peritumoral Tissue

Since the early 1970s, when Folkman proposed angiogenesis as a fundamental process for tumor growth [110], many groups across the world focused on the development of potential anti-angiogenic therapies to treat different human tumors. In 2004, the first approved anti-angiogenesis drug known as bevacizumab (Avastin) was introduced, a monoclonal antibody against VEGF approved for the treatment of metastatic colorectal cancer. Solid tumors would not have significant chances to growth in the absence of a local blood supply, which is necessary to provide sufficient diffusion of oxygen and nutrients to sustain tissue viability [111,112,113]. In a deficit of blood supply, hypoxia stimulates hypoxia-inducible factors and VEGF secretion in both tumor cells and tumor-associated stromal cells. Release of specific pro-angiogenic factors stimulates new blood vessel development into the tumor. As a result, tumor-induced angiogenesis provides crucial nourishment to tumor cells, allowing the neoplastic mass to expand, invading the surrounding tissues and eventually spreading from the original site into metastases, establishing secondary areas of proliferation [114]. Neoangiogenesis in the tumor surrounding areas was explored by Sica et al. [8] by evaluating the endothelial activation and the presence of microvessels. In particular, the expression of nestin and CD105 in the vessel wall was analyzed and the micro-vessel density (MVD) was determined at a distance <1 cm and between 1 and 3.5 cm from the macroscopic tumor border. Nestin is presumably involved in the rapid turnover of the ECs. The results of this study clearly indicate that neoangiogenesis occurs in the peritumoral tissue with the intimate involvement of pericytes. Moreover, the MVD in the tissue located at a greater distance from the tumor margin correlates with the median patient survival time. It was demonstrated that a sub-population of GBM stem-like cells (GSCs) can trigger the formation of a functional vasculature [115,116], which is crucial for the tumor to progress. Based on this metabolic need of tumor cells, many researchers struggled to develop angiogenesis inhibitors to block tumor-induced neoangiogenesis [117,118]. As revealed by routine histological evaluation, GBM shows an extensive neovascularization, [119,120], with extremely disorganized, high permeable, and tortuous tumor vessels with an altered basement membrane [121]. Distinct angiogenesis mechanisms, including sprouting angiogenesis, recruitment of endothelial progenitor cells, intussusceptive angiogenesis, and vascular mimicry, are involved in tumor neovascularization [122] including in gliomas [120]. More recently, our group focused on the expression of different factors and receptors involved in the angiogenic process both in the tissue and in CSCs isolated from patients that had previously undergone surgery. In particular, tissue was derived at a distance <1 cm from the macroscopic tumor border. CSCs were obtained from the GBM (GCSCs) and from the same area previously indicated (PCSCs). Immunohistochemistry demonstrated the expression of angiogenetic markers in both GBM and peritumoral tissue. Interestingly, both GCSCs and PCSCs were able to stimulate the angiogenic response of human ECs [12]. To this regard, in hepatocellular carcinoma, peritumoral ECs show a higher proliferation rate compared with tumor ECs [123]. It is, therefore, conceivable that peculiar modifications and molecular determinants occurring in the peritumoral tissue may play a crucial role in the recurrence of GBM. 

## 6. Cancer Stem Cells in GBM and Peritumoral Tissue

Undeniable characteristics of GBMs are the high degree of cellular and genetic heterogeneity and the strong talent to invade other tissues [124]. Despite the aggressive standard therapeutic approaches, which include surgical resection and radiotherapy with concomitant chemotherapy, the prognosis remains poor [125]. These unfavorable outcomes are attributable to GBM stem cells (GSCs), which comprise a small sub-population of tumor cells that have several phenotypic and functional similarities with normal neural stem cells (NSCs) [126,127,128,129]. NSCs are primarily located in the sub-ventricular zone of the brain, which is a common site of origin for glioma [130]. Several studies demonstrate that pathways playing a role in NSCs differentiation, including activation of Protein kinase B, RAS/ERK, polycomb ring finger oncogene (BMI-1), NOTCH, and WNT, frequently show genomic alteration or aberrant activation in GBM. In addition, GSCs express many of the characteristic markers of NSCs, including CD133, SOX2, and nestin, and demonstrate upregulation of glial fibrillary acidic protein during differentiation to an astrocytic lineage [128]. Despite the evidence demonstrating shared signaling pathways and biomarker expression between NSCs and GSCs, and although emerging studies support the hypothesis that NSCs are the target cells where tumor-initiating genomic alterations may occur, it is still unclear whether GSCs originate from mutated NSCs or if they derive from mature glial cells that dedifferentiated and acquired the ability to self-renew [131]. GSCs were first identified in 2002 [132], and further investigation demonstrated that they contribute to tumor maintenance and propagation [129,133,134], as well as resistance to therapy [135,136]. In vitro, GSCs form neurospheres [137], show self-renewal capabilities [138], and, when injected in immunosuppressed mice, generate a tumor that resembles the parental one in terms of antigen expression and histological organization [129,133,139]. The identification and isolation of putative GSCs rely on the differential stem-cell surface marker expression profile. CD133, a transmembrane glycoprotein, is the most widely recognized and reliable stemness biomarker. In fact, CD133-positive cells are able to grow in neurospheres and recapitulate human tumors after injection in animal models [129]. In addition, CD133-positive cells show higher resistance to radiation and chemotherapy, a reduced level of apoptosis, and an increased colony-forming efficiency when compared to CD133-negative cells [136]. Despite the evidence outlining its crucial relationship with GSCs, CD133 is not a universal marker for identifying GSCs, and other factors may collaborate with CD133 to increase the stemness of GSCs. Experimental evidence demonstrated that SOX2 expression contributes to GBM stem-cell potency by regulating CD133 levels in CD133-positive GBM cells [140,141]. Interestingly, targeting SOX2 by RNA interference (RNAi) strongly affects tumor-initiating ability, as well as drug resistance, of CD133-positive GBM cells, suggesting a key role for SOX2 in the regulation of tumorigenicity in these cells [142]. Nestin is a crucial factor in different types of cancer [143,144,145] and, in particular, in GBM [129,134,136,143,146,147]. Increased levels of nestin expression are found in higher-grade gliomas and in patients with lower survival rates [148]. In recent years, a number of molecules were identified as putative markers used in order to enrich GSCs, including CD44 [149], CD49f (integrin α6) [150], Musashi [151,152], Nanog [153,154], and Oct4 [155]. Nevertheless, the quest for a universal GSC marker is still open [132]. Indeed, GSCs are placed in a specific microenvironment known as the “niche”, where their stemness is maintained. The complex interactions between the GSCs and the numerous components of the niche may regulate several processes including tumor initiation, survival, and invasion, ultimately affecting the response to therapy [149,150,156]. Therefore, any alteration of the interplay between the GSCs and the cells of their niche may represent important determinants of functional tumor microenvironment favoring cancer development [157]. Among the well-established tumor niches for GBM are the perivascular niches [149,158,159], which drastically influence the behavior of resident GSCs. ECs can interact specifically with nestin CD133-positive GSCs located in the proximity of capillaries, and produce a variety of growth factors participating in the maintenance of GSC self-renewing and undifferentiated state [156,160,161,162]. GSCs, in turn, produce VEGF and a variety of cytokines and chemokines, some of which are known to activate ECs [159,163,164], suggesting that GSCs may regulate tumor angiogenesis [150,156]. In addition, recent studies showed that GSCs may transdifferentiate into ECs or pericytes, creating their own vascular niches [115,116,150,165]. In addition to an aberrant vasculature, GBM contains areas of intra-tumor necrosis that are typically associated with tissue hypoxia. Actually, the hypercellular and highly hypoxic area surrounding the necrotic foci, called the pseudopalisades, plays a key role in the maintenance and propagation of CD133-positive GSCs [166,167]. Although it may negatively affect tumor cell growth, exposure to hypoxia induces malignant progression and aggressiveness, and it leads to increased resistance to therapy and poor prognosis. Hypoxia upregulates VEGF expression in GSCs and increases angiogenesis [120]. GBM shows an aggressive behavior, invading adjacent healthy tissue and making surgical resection challenging. Indeed, the presence of a pool of invasive cells was found in the tissue adjacent to the resection margin or within 2 to 3 cm of the resection cavity [97]. Notably, the presence of infiltrative tumor cells in the peritumoral tissue that were not detectable at histological analysis was reported [9]. These findings were further confirmed by the identification of CD133- and nestin-positive cells, as well as the expression of progenitor/stem-cell markers GD3 ganglioside and NG2 proteoglycan and angiogenesis-related factors (VEGF, VEGF receptors 1 and 2, Hypoxia-inducible factor-1α and -2α), in both GBM and in the peritumoral tissue, where this expression was also detected in apparently normal cells [8,12,13]. Since cells with stem-like features were identified in the peritumoral tissue, it is possible that they may play a role in tumor recurrence occurring in this area. Nevertheless, CSCs derived either from the tumor mass or the peritumoral tissue at least 2 cm away from it show different tumorigenic potential and genetic characteristics [168]. In addition, the same behavior is shown by tumor-initiating cells derived from the tumor margin with respect to those isolated from the tumor mass [169]. Recent studies of our group were performed on pairs of CSCs derived from the GBM core (GCSCs) or the peritumoral tissue (PCSCs) at a distance ≤1 cm from the macroscopic tumor border, isolated from the same patients. The results of these studies demonstrated that GCSCs and PCSCs show different behaviors and molecular features in terms of proliferative potential, ultrastructure, and expression of stem-cell markers, c-Met, Mitogen-activated protein kinases, H19 lncRNA, and miR-675-5p. These data suggest that PCSCs are less aggressive compared to GCSCs [14]. However, PCSCs subjected to treatment with temozolomide, alone or in combination with adjuvant molecules, seemed more resistant to therapy than GCSCs [15]. In addition, it was reported that the aptitude of GBM stem-like or initiating cells to invade the surrounding tissue is linked to the upregulation of αVβ3 integrin and diminished expression of p27, upstream regulators of the RhoA family members [170]. These studies would suggest the existence of precise mechanisms regulating the motility of GBM stem-like cells and those that infiltrate from the peritumoral region into the brain parenchyma. 

## 7. Conclusions and Future Perspectives

Despite radical surgical resection followed by aggressive chemo- and radiotherapy, the prognosis of GBM remains dismal. The continuous effort to identify novel potential molecular targets for the development of effective clinical therapies is yet to lead to significant improvements in the survival rate, and the majority of patients do not survive beyond three years. In addition, the great histological heterogeneity of GBM and the multiplicity of underlying molecular mechanisms are the main reasons for resistance against standard radio- and chemotherapy, making the experimental investigation of GBM extremely challenging. This molecular and functional complexity of GBM spurred investigators to pay specific attention to alternative strategies, to more effective and less toxic therapy, such as immunotherapy. Immune checkpoint therapy aims to overstep the tumor-induced tolerance, through the reversal of T-cell exhaustion and restoration of anti-tumor immunity, and several clinical trials are currently ongoing on brain tumor patients. The development of specific vaccines for GBM is under investigation, seeking a personalized treatment for GBM patients. Even miRNAs, whose dysregulation plays a leading role in mechanisms of glioma progression, may represent promising targets for new therapeutic approaches in GBM. Moreover, further studies of the two stem-cell-like populations, residing in the tumor and in the peritumoral tissue, are crucial for understanding GBM propagation and drug resistance, and could shed light on potential therapeutic targets. Finally, on the basis of the recent findings, we believe that further characterization of the molecular alterations occurring in the peritumoral tissue of GBM, as well as a definition of the role played by CSCs found in this tissue, may be of great interest to identify new molecular targets and to facilitate the development of personalized therapies.

## References

[1] Sathornsumetee S., Rich J.N., Reardon D.A. (2007). Diagnosis and treatment of high-grade astrocytoma. Neurol. Clin..

[2] Bush N.A., Chang S.M., Berger M.S. (2017). Current and future strategies for treatment of glioma. Neurosurg. Rev..

[3] Walker M.D., Alexander E., Hunt W.E., MacCarty C.S., Mahaley M.S., Mealey J., Norrell H.A., Owens G., Ransohoff J., Wilson C.B. (1978). Evaluation of BCNU and/or radiotherapy in the treatment of anaplastic gliomas. A cooperative clinical trial. J. Neurosurg..

[4] Stupp R., Mason W.P., van den Bent M.J., Weller M., Fisher B., Taphoorn M.J., Belanger K., Brandes A.A., Marosi C., Bogdahn U. (2005). Radiotherapy plus concomitant and adjuvant temozolomide for glioblastoma. N. Engl. J. Med..

[5] Stupp R., Hegi M.E., Mason W.P., van den Bent M.J., Taphoorn M.J., Janzer R.C., Ludwin S.K., Allgeier A., Fisher B., Belanger K. (2009). Effects of radiotherapy with concomitant and adjuvant temozolomide versus radiotherapy alone on survival in glioblastoma in a randomised phase III study: 5-year analysis of the EORTC-NCIC trial. Lancet Oncol..

[6] Lama G., Mangiola A., Anile C., Sabatino G., De Bonis P., Lauriola L., Giannitelli C., La Torre G., Jhanwar-Uniyal M., Sica G. (2007). Activated ERK1/2 expression in glioblastoma multiforme and in peritumor tissue. Int. J. Oncol..

[7] Mangiola A., Lama G., Giannitelli C., De Bonis P., Anile C., Lauriola L., La Torre G., Sabatino G., Maira G., Jhanwar-Uniyal M. (2007). Stem cell marker nestin and c-Jun NH2-terminal kinases in tumor and peritumor areas of glioblastoma multiforme: Possible prognostic implications. Clin. Cancer Res..

[8] Sica G., Lama G., Anile C., Geloso M.C., La Torre G., De Bonis P., Maira G., Lauriola L., Jhanwar-Uniyal M., Mangiola A. (2011). Assessment of angiogenesis by CD105 and nestin expression in peritumor tissue of glioblastoma. Int. J. Oncol..

[9] Mangiola A., Saulnier N., De Bonis P., Orteschi D., Sica G., Lama G., Pettorini B.L., Sabatino G., Zollino M., Lauriola L. (2013). Gene expression profile of glioblastoma peritumoral tissue: An ex vivo study. PLoS ONE.

[10] Cenciarelli C., Marei H.E., Zonfrillo M., Pierimarchi P., Paldino E., Casalbore P., Felsani A., Vescovi A.L., Maira G., Mangiola A. (2014). PDGF receptor alpha inhibition induces apoptosis in glioblastoma cancer stem cells refractory to anti-Notch and anti-EGFR treatment. Mol. Cancer.

[11] Lemee J.M., Clavreul A., Menei P. (2015). Intratumoral heterogeneity in glioblastoma: Don’t forget the peritumoral brain zone. Neuro-Oncology.

[12] D’Alessio A., Proietti G., Lama G., Biamonte F., Lauriola L., Moscato U., Vescovi A., Mangiola A., Angelucci C., Sica G. (2016). Analysis of angiogenesis related factors in glioblastoma, peritumoral tissue and their derived cancer stem cells. Oncotarget.

[13] Lama G., Mangiola A., Proietti G., Colabianchi A., Angelucci C., D’Alessio A., De Bonis P., Geloso M.C., Lauriola L., Binda E. (2016). Progenitor/Stem Cell Markers in Brain Adjacent to Glioblastoma: GD3 Ganglioside and NG2 Proteoglycan Expression. J. Neuropathol. Exp. Neurol..

[14] Angelucci C., D’Alessio A., Lama G., Binda E., Mangiola A., Vescovi A.L., Proietti G., Masuelli L., Bei R., Fazi B. (2018). Cancer stem cells from peritumoral tissue of glioblastoma multiforme: the possible missing link between tumor development and progression. Oncotarget.

[15] Scicchitano B.M., Sorrentino S., Proietti G., Lama G., Dobrowolny G., Catizone A., Binda E., Larocca L.M., Sica G. (2018). Levetiracetam enhances the temozolomide effect on glioblastoma stem cell proliferation and apoptosis. Cancer Cell Int..

[16] Louveau A., Smirnov I., Keyes T.J., Eccles J.D., Rouhani S.J., Peske J.D., Derecki N.C., Castle D., Mandell J.W., Lee K.S. (2015). Structural and functional features of central nervous system lymphatic vessels. Nature.

[17] Louis D.N., Perry A., Reifenberger G., von Deimling A., Figarella-Branger D., Cavenee W.K., Ohgaki H., Wiestler O.D., Kleihues P., Ellison D.W. (2016). The 2016 World Health Organization Classification of Tumors of the Central Nervous System: A summary. Acta Neuropathol..

[18] Ceccarelli M., Barthel F.P., Malta T.M., Sabedot T.S., Salama S.R., Murray B.A., Morozova O., Newton Y., Radenbaugh A., Pagnotta S.M. (2016). Molecular Profiling Reveals Biologically Discrete Subsets and Pathways of Progression in Diffuse Glioma. Cell.

[19] Verhaak R.G., Hoadley K.A., Purdom E., Wang V., Qi Y., Wilkerson M.D., Miller C.R., Ding L., Golub T., Mesirov J.P. (2010). Integrated genomic analysis identifies clinically relevant subtypes of glioblastoma characterized by abnormalities in PDGFRA, IDH1, EGFR, and NF1. Cancer Cell.

[20] Ohgaki H., Kleihues P. (2013). The definition of primary and secondary glioblastoma. Clin. Cancer Res..

[21] Parker N.R., Khong P., Parkinson J.F., Howell V.M., Wheeler H.R. (2015). Molecular heterogeneity in glioblastoma: Potential clinical implications. Front. Oncol..

[22] Qazi M.A., Vora P., Venugopal C., Sidhu S.S., Moffat J., Swanton C., Singh S.K. (2017). Intratumoral heterogeneity: Pathways to treatment resistance and relapse in human glioblastoma. Ann. Oncol..

[23] Daniel P.M., Filiz G., Tymms M.J., Ramsay R.G., Kaye A.H., Stylli S.S., Mantamadiotis T. (2018). Intratumor MAPK and PI3K signaling pathway heterogeneity in glioblastoma tissue correlates with CREB signaling and distinct target gene signatures. Exp. Mol. Pathol..

[24] Lu C., Ward P.S., Kapoor G.S., Rohle D., Turcan S., Abdel-Wahab O., Edwards C.R., Khanin R., Figueroa M.E., Melnick A. (2012). IDH mutation impairs histone demethylation and results in a block to cell differentiation. Nature.

[25] Molenaar R.J., Maciejewski J.P., Wilmink J.W., van Noorden C.J.F. (2018). Wild-type and mutated IDH1/2 enzymes and therapy responses. Oncogene.

[26] Turcan S., Rohle D., Goenka A., Walsh L.A., Fang F., Yilmaz E., Campos C., Fabius A.W., Lu C., Ward P.S. (2012). IDH1 mutation is sufficient to establish the glioma hypermethylator phenotype. Nature.

[27] Wesseling P., Capper D. (2018). WHO 2016 Classification of gliomas. Neuropathol. Appl. Neurobiol..

[28] Wick W., Weller M., van den Bent M., Sanson M., Weiler M., von Deimling A., Plass C., Hegi M., Platten M., Reifenberger G. (2014). MGMT testing--the challenges for biomarker-based glioma treatment. Nat. Rev. Neurol..

[29] Hegi M.E., Diserens A.C., Gorlia T., Hamou M.F., de Tribolet N., Weller M., Kros J.M., Hainfellner J.A., Mason W., Mariani L. (2005). MGMT gene silencing and benefit from temozolomide in glioblastoma. N. Engl. J. Med..

[30] Brennan C.W., Verhaak R.G., McKenna A., Campos B., Noushmehr H., Salama S.R., Zheng S., Chakravarty D., Sanborn J.Z., Berman S.H. (2013). The somatic genomic landscape of glioblastoma. Cell.

[31] Wong A.J., Ruppert J.M., Bigner S.H., Grzeschik C.H., Humphrey P.A., Bigner D.S., Vogelstein B. (1992). Structural alterations of the epidermal growth factor receptor gene in human gliomas. Proc. Natl. Acad. Sci. USA.

[32] Burnet F.M. (1970). The concept of immunological surveillance. Prog. Exp. Tumor Res..

[33] Vesely M.D., Kershaw M.H., Schreiber R.D., Smyth M.J. (2011). Natural innate and adaptive immunity to cancer. Annu. Rev. Immunol..

[34] Morantz R.A., Shain W., Cravioto H. (1978). Immune surveillance and tumors of the nervous system. J. Neurosurg..

[35] Wraith D.C., Nicholson L.B. (2012). The adaptive immune system in diseases of the central nervous system. J. Clin. Investig..

[36] Muldoon L.L., Alvarez J.I., Begley D.J., Boado R.J., Del Zoppo G.J., Doolittle N.D., Engelhardt B., Hallenbeck J.M., Lonser R.R., Ohlfest J.R. (2013). Immunologic privilege in the central nervous system and the blood-brain barrier. J. Cereb. Blood Flow Metab..

[37] Streilein J.W. (1993). Immune privilege as the result of local tissue barriers and immunosuppressive microenvironments. Curr. Opin. Immunol..

[38] Serlin Y., Shelef I., Knyazer B., Friedman A. (2015). Anatomy and physiology of the blood-brain barrier. Semin. Cell Dev. Biol..

[39] Suter T., Biollaz G., Gatto D., Bernasconi L., Herren T., Reith W., Fontana A. (2003). The brain as an immune privileged site: Dendritic cells of the central nervous system inhibit T cell activation. Eur. J. Immunol..

[40] Lukic I.K., Gluncic V., Ivkic G., Hubenstorf M., Marusic A. (2003). Virtual dissection: A lesson from the 18th century. Lancet.

[41] Aspelund A., Antila S., Proulx S.T., Karlsen T.V., Karaman S., Detmar M., Wiig H., Alitalo K. (2015). A dural lymphatic vascular system that drains brain interstitial fluid and macromolecules. J. Exp. Med..

[42] Rua R., McGavern D.B. (2018). Advances in Meningeal Immunity. Trends Mol. Med..

[43] Domingues P., Gonzalez-Tablas M., Otero A., Pascual D., Miranda D., Ruiz L., Sousa P., Ciudad J., Goncalves J.M., Lopes M.C. (2016). Tumor infiltrating immune cells in gliomas and meningiomas. Brain Behav. Immun..

[44] Huang B., Zhang H., Gu L., Ye B., Jian Z., Stary C., Xiong X. (2017). Advances in Immunotherapy for Glioblastoma Multiforme. J. Immunol. Res..

[45] Gieryng A., Pszczolkowska D., Walentynowicz K.A., Rajan W.D., Kaminska B. (2017). Immune microenvironment of gliomas. Lab. Investig..

[46] Pereira M.B., Barros L.R.C., Bracco P.A., Vigo A., Boroni M., Bonamino M.H., Lenz G. (2018). Transcriptional characterization of immunological infiltrates and their relation with glioblastoma patients overall survival. Oncoimmunology.

[47] Razavi S.M., Lee K.E., Jin B.E., Aujla P.S., Gholamin S., Li G. (2016). Immune Evasion Strategies of Glioblastoma. Front. Surg..

[48] Hambardzumyan D., Gutmann D.H., Kettenmann H. (2016). The role of microglia and macrophages in glioma maintenance and progression. Nat. Neurosci..

[49] Li Q., Barres B.A. (2018). Microglia and macrophages in brain homeostasis and disease. Nat. Rev. Immunol..

[50] Chen Z., Feng X., Herting C.J., Garcia V.A., Nie K., Pong W.W., Rasmussen R., Dwivedi B., Seby S., Wolf S.A. (2017). Cellular and Molecular Identity of Tumor-Associated Macrophages in Glioblastoma. Cancer Res..

[51] Abounader R., Laterra J. (2005). Scatter factor/hepatocyte growth factor in brain tumor growth and angiogenesis. Neuro Oncol..

[52] Ellert-Miklaszewska A., Wisniewski P., Kijewska M., Gajdanowicz P., Pszczolkowska D., Przanowski P., Dabrowski M., Maleszewska M., Kaminska B. (2016). Tumour-processed osteopontin and lactadherin drive the protumorigenic reprogramming of microglia and glioma progression. Oncogene.

[53] Szulzewsky F., Pelz A., Feng X., Synowitz M., Markovic D., Langmann T., Holtman I.R., Wang X., Eggen B.J., Boddeke H.W. (2015). Glioma-associated microglia/macrophages display an expression profile different from M1 and M2 polarization and highly express Gpnmb and Spp1. PLoS ONE.

[54] Da Fonseca A.C., Amaral R., Garcia C., Geraldo L.H., Matias D., Lima F.R. (2016). Microglia in Cancer: For Good or for Bad?. Adv. Exp. Med. Biol..

[55] Hattermann K., Sebens S., Helm O., Schmitt A.D., Mentlein R., Mehdorn H.M., Held-Feindt J. (2014). Chemokine expression profile of freshly isolated human glioblastoma-associated macrophages/microglia. Oncol. Rep..

[56] Markovic D.S., Glass R., Synowitz M., Rooijen N., Kettenmann H. (2005). Microglia stimulate the invasiveness of glioma cells by increasing the activity of metalloprotease-2. J. Neuropathol. Exp. Neurol..

[57] Weathers S.P., de Groot J. (2015). VEGF Manipulation in Glioblastoma. Oncology.

[58] Zhang I., Alizadeh D., Liang J., Zhang L., Gao H., Song Y., Ren H., Ouyang M., Wu X., D’Apuzzo M. (2016). Characterization of Arginase Expression in Glioma-Associated Microglia and Macrophages. PLoS ONE.

[59] Shi Y., Ping Y.F., Zhou W., He Z.C., Chen C., Bian B.S., Zhang L., Chen L., Lan X., Zhang X.C. (2017). Tumour-associated macrophages secrete pleiotrophin to promote PTPRZ1 signalling in glioblastoma stem cells for tumour growth. Nat. Commun..

[60] Roesch S., Rapp C., Dettling S., Herold-Mende C. (2018). When Immune Cells Turn Bad-Tumor-Associated Microglia/Macrophages in Glioma. Int. J. Mol. Sci..

[61] Haile L.A., Greten T.F., Korangy F. (2012). Immune suppression: The hallmark of myeloid derived suppressor cells. Immunol. Investig..

[62] Pilatova K., Bencsikova B., Demlova R., Valik D., Zdrazilova-Dubska L. (2018). Myeloid-derived suppressor cells (MDSCs) in patients with solid tumors: Considerations for granulocyte colony-stimulating factor treatment. Cancer Immunol. Immunother..

[63] Li H., Han Y., Guo Q., Zhang M., Cao X. (2009). Cancer-expanded myeloid-derived suppressor cells induce anergy of NK cells through membrane-bound TGF-beta 1. J. Immunol..

[64] Srivastava M.K., Sinha P., Clements V.K., Rodriguez P., Ostrand-Rosenberg S. (2010). Myeloid-derived suppressor cells inhibit T-cell activation by depleting cystine and cysteine. Cancer Res..

[65] Waldron T.J., Quatromoni J.G., Karakasheva T.A., Singhal S., Rustgi A.K. (2013). Myeloid derived suppressor cells: Targets for therapy. Oncoimmunology.

[66] Zhang S., Ma X., Zhu C., Liu L., Wang G., Yuan X. (2016). The Role of Myeloid-Derived Suppressor Cells in Patients with Solid Tumors: A Meta-Analysis. PLoS ONE.

[67] Gielen P.R., Schulte B.M., Kers-Rebel E.D., Verrijp K., Bossman S.A., Ter Laan M., Wesseling P., Adema G.J. (2016). Elevated levels of polymorphonuclear myeloid-derived suppressor cells in patients with glioblastoma highly express S100A8/9 and arginase and suppress T cell function. Neuro Oncol..

[68] Dubinski D., Wolfer J., Hasselblatt M., Schneider-Hohendorf T., Bogdahn U., Stummer W., Wiendl H., Grauer O.M. (2016). CD4+ T effector memory cell dysfunction is associated with the accumulation of granulocytic myeloid-derived suppressor cells in glioblastoma patients. Neuro-Oncology.

[69] Lohr J., Ratliff T., Huppertz A., Ge Y., Dictus C., Ahmadi R., Grau S., Hiraoka N., Eckstein V., Ecker R.C. (2011). Effector T-cell infiltration positively impacts survival of glioblastoma patients and is impaired by tumor-derived TGF-beta. Clin. Cancer Res..

[70] Rutledge W.C., Kong J., Gao J., Gutman D.A., Cooper L.A., Appin C., Park Y., Scarpace L., Mikkelsen T., Cohen M.L. (2013). Tumor-infiltrating lymphocytes in glioblastoma are associated with specific genomic alterations and related to transcriptional class. Clin. Cancer Res..

[71] Ooi Y.C., Tran P., Ung N., Thill K., Trang A., Fong B.M., Nagasawa D.T., Lim M., Yang I. (2014). The role of regulatory T-cells in glioma immunology. Clin. Neurol. Neurosurg..

[72] Papamichail M., Perez S.A., Gritzapis A.D., Baxevanis C.N. (2004). Natural killer lymphocytes: Biology, development, and function. Cancer Immunol. Immunother..

[73] Chan C.J., Smyth M.J., Martinet L. (2014). Molecular mechanisms of natural killer cell activation in response to cellular stress. Cell Death Differ..

[74] Jung T.Y., Choi Y.D., Kim Y.H., Lee J.J., Kim H.S., Kim J.S., Kim S.K., Jung S., Cho D. (2013). Immunological characterization of glioblastoma cells for immunotherapy. Anticancer Res..

[75] Avril T., Vauleon E., Hamlat A., Saikali S., Etcheverry A., Delmas C., Diabira S., Mosser J., Quillien V. (2012). Human glioblastoma stem-like cells are more sensitive to allogeneic NK and T cell-mediated killing compared with serum-cultured glioblastoma cells. Brain Pathol..

[76] Castriconi R., Daga A., Dondero A., Zona G., Poliani P.L., Melotti A., Griffero F., Marubbi D., Spaziante R., Bellora F. (2009). NK cells recognize and kill human glioblastoma cells with stem cell-like properties. J. Immunol..

[77] Eisele G., Wischhusen J., Mittelbronn M., Meyermann R., Waldhauer I., Steinle A., Weller M., Friese M.A. (2006). TGF-beta and metalloproteinases differentially suppress NKG2D ligand surface expression on malignant glioma cells. Brain.

[78] Zhang X., Rao A., Sette P., Deibert C., Pomerantz A., Kim W.J., Kohanbash G., Chang Y., Park Y., Engh J. (2016). IDH mutant gliomas escape natural killer cell immune surveillance by downregulation of NKG2D ligand expression. Neuro-Oncology.

[79] Bartel D.P. (2004). MicroRNAs: Genomics, biogenesis, mechanism, and function. Cell.

[80] Chan J.A., Krichevsky A.M., Kosik K.S. (2005). MicroRNA-21 is an antiapoptotic factor in human glioblastoma cells. Cancer Res..

[81] Ciafre S.A., Galardi S., Mangiola A., Ferracin M., Liu C.G., Sabatino G., Negrini M., Maira G., Croce C.M., Farace M.G. (2005). Extensive modulation of a set of microRNAs in primary glioblastoma. Biochem. Biophys. Res. Commun..

[82] Singh S.K., Vartanian A., Burrell K., Zadeh G. (2012). A microRNA Link to Glioblastoma Heterogeneity. Cancers.

[83] Banelli B., Forlani A., Allemanni G., Morabito A., Pistillo M.P., Romani M. (2017). MicroRNA in Glioblastoma: An Overview. Int. J. Genom..

[84] Calin G.A., Dumitru C.D., Shimizu M., Bichi R., Zupo S., Noch E., Aldler H., Rattan S., Keating M., Rai K. (2002). Frequent deletions and down-regulation of micro- RNA genes miR15 and miR16 at 13q14 in chronic lymphocytic leukemia. Proc. Natl. Acad. Sci. USA.

[85] Lujambio A., Lowe S.W. (2012). The microcosmos of cancer. Nature.

[86] Xia H., Yan Y., Hu M., Wang Y., Wang Y., Dai Y., Chen J., Di G., Chen X., Jiang X. (2013). MiR-218 sensitizes glioma cells to apoptosis and inhibits tumorigenicity by regulating ECOP-mediated suppression of NF-kappaB activity. Neuro-Oncology.

[87] Conti A., Aguennouz M., La Torre D., Tomasello C., Cardali S., Angileri F.F., Maio F., Cama A., Germano A., Vita G. (2009). miR-21 and 221 upregulation and miR-181b downregulation in human grade II-IV astrocytic tumors. J. Neurooncol..

[88] Medina R., Zaidi S.K., Liu C.G., Stein J.L., van Wijnen A.J., Croce C.M., Stein G.S. (2008). MicroRNAs 221 and 222 bypass quiescence and compromise cell survival. Cancer Res..

[89] Gabriely G., Wurdinger T., Kesari S., Esau C.C., Burchard J., Linsley P.S., Krichevsky A.M. (2008). MicroRNA 21 promotes glioma invasion by targeting matrix metalloproteinase regulators. Mol. Cell Biol..

[90] Sasayama T., Nishihara M., Kondoh T., Hosoda K., Kohmura E. (2009). MicroRNA-10b is overexpressed in malignant glioma and associated with tumor invasive factors, uPAR and RhoC. Int. J. Cancer.

[91] Godlewski J., Nowicki M.O., Bronisz A., Williams S., Otsuki A., Nuovo G., Raychaudhury A., Newton H.B., Chiocca E.A., Lawler S. (2008). Targeting of the Bmi-1 oncogene/stem cell renewal factor by microRNA-128 inhibits glioma proliferation and self-renewal. Cancer Res..

[92] Deng Y., Zhu G., Luo H., Zhao S. (2016). MicroRNA-203 As a Stemness Inhibitor of Glioblastoma Stem Cells. Mol. Cells.

[93] Lopez-Bertoni H., Lal B., Michelson N., Guerrero-Cazares H., Quinones-Hinojosa A., Li Y., Laterra J. (2016). Epigenetic modulation of a miR-296-5p:HMGA1 axis regulates Sox2 expression and glioblastoma stem cells. Oncogene.

[94] Sun Y.C., Wang J., Guo C.C., Sai K., Wang J., Chen F.R., Yang Q.Y., Chen Y.S., Wang J., To T.S. (2014). MiR-181b sensitizes glioma cells to teniposide by targeting MDM2. BMC Cancer.

[95] Shi L., Wan Y., Sun G., Gu X., Qian C., Yan W., Zhang S., Pan T., Wang Z., You Y. (2012). Functional differences of miR-125b on the invasion of primary glioblastoma CD133-negative cells and CD133-positive cells. Neuromol. Med..

[96] Beyer S., Fleming J., Meng W., Singh R., Haque S.J., Chakravarti A. (2017). The Role of miRNAs in Angiogenesis, Invasion and Metabolism and Their Therapeutic Implications in Gliomas. Cancers.

[97] Burger P.C., Dubois P.J., Schold S.C., Smith K.R., Odom G.L., Crafts D.C., Giangaspero F. (1983). Computerized tomographic and pathologic studies of the untreated, quiescent, and recurrent glioblastoma multiforme. J. Neurosurg..

[98] Buckingham S.C., Campbell S.L., Haas B.R., Montana V., Robel S., Ogunrinu T., Sontheimer H. (2011). Glutamate release by primary brain tumors induces epileptic activity. Nat. Med..

[99] Roy S., Sarkar C. (1989). Ultrastructural study of micro-blood vessels in human brain tumors and peritumoral tissue. J. Neurooncol..

[100] Ito U., Reulen H.J., Huber P. (1986). Spatial and quantitative distribution of human peritumoural brain oedema in computerized tomography. Acta Neurochir..

[101] Papadopoulos M.C., Saadoun S., Davies D.C., Bell B.A. (2001). Emerging molecular mechanisms of brain tumour oedema. Br. J. Neurosurg..

[102] Bakshi A., Nag T.C., Wadhwa S., Mahapatra A.K., Sarkar C. (1998). The expression of nitric oxide synthases in human brain tumours and peritumoral areas. J. Neurol. Sci..

[103] Eyler C.E., Wu Q., Yan K., MacSwords J.M., Chandler-Militello D., Misuraca K.L., Lathia J.D., Forrester M.T., Lee J., Stamler J.S. (2011). Glioma stem cell proliferation and tumor growth are promoted by nitric oxide synthase-2. Cell.

[104] Melani A., De Micheli E., Pinna G., Alfieri A., Corte L.D., Pedata F. (2003). Adenosine extracellular levels in human brain gliomas: An intraoperative microdialysis study. Neurosci. Lett..

[105] Bauer A., Langen K.J., Bidmon H., Holschbach M.H., Weber S., Olsson R.A., Coenen H.H., Zilles K. (2005). 18F-CPFPX PET identifies changes in cerebral A1 adenosine receptor density caused by glioma invasion. J. Nucl. Med..

[106] Dehnhardt M., Zoriy M.V., Khan Z., Reifenberger G., Ekstrom T.J., Sabine Becker J., Zilles K., Bauer A. (2008). Element distribution is altered in a zone surrounding human glioblastoma multiforme. J. Trace Elem. Med. Biol. Organ. Soc. Miner. Trace Elem..

[107] Cubillos S., Obregon F., Vargas M.F., Salazar L.A., Lima L. (2006). Taurine concentration in human gliomas and meningiomas: Tumoral, peritumoral, and extratumoral tissue. Adv. Exp. Med. Biol..

[108] Haybaeck J., Obrist P., Schindler C.U., Spizzo G., Doppler W. (2007). STAT-1 expression in human glioblastoma and peritumoral tissue. Anticancer Res..

[109] Mangiola A., de Bonis P., Maira G., Balducci M., Sica G., Lama G., Lauriola L., Anile C. (2008). Invasive tumor cells and prognosis in a selected population of patients with glioblastoma multiforme. Cancer.

[110] Folkman J. (1971). Tumor angiogenesis: Therapeutic implications. N. Engl. J. Med..

[111] Hanahan D., Folkman J. (1996). Patterns and emerging mechanisms of the angiogenic switch during tumorigenesis. Cell.

[112] Carmeliet P. (2005). Angiogenesis in life, disease and medicine. Nature.

[113] Jain R.K., Au P., Tam J., Duda D.G., Fukumura D. (2005). Engineering vascularized tissue. Nat. Biotechnol..

[114] Folkman J. (2002). Role of angiogenesis in tumor growth and metastasis. Semin. Oncol..

[115] Ricci-Vitiani L., Pallini R., Biffoni M., Todaro M., Invernici G., Cenci T., Maira G., Parati E.A., Stassi G., Larocca L.M. (2010). Tumour vascularization via endothelial differentiation of glioblastoma stem-like cells. Nature.

[116] Wang R., Chadalavada K., Wilshire J., Kowalik U., Hovinga K.E., Geber A., Fligelman B., Leversha M., Brennan C., Tabar V. (2010). Glioblastoma stem-like cells give rise to tumour endothelium. Nature.

[117] Cook K.M., Figg W.D. (2010). Angiogenesis inhibitors: Current strategies and future prospects. CA Cancer J. Clin..

[118] El-Kenawi A.E., El-Remessy A.B. (2013). Angiogenesis inhibitors in cancer therapy: Mechanistic perspective on classification and treatment rationales. Br. J. Pharm..

[119] Huse J.T., Holland E.C. (2010). Targeting brain cancer: Advances in the molecular pathology of malignant glioma and medulloblastoma. Nat. Rev. Cancer.

[120] Hardee M.E., Zagzag D. (2012). Mechanisms of glioma-associated neovascularization. Am. J. Pathol..

[121] Jain R.K., di Tomaso E., Duda D.G., Loeffler J.S., Sorensen A.G., Batchelor T.T. (2007). Angiogenesis in brain tumours. Nat. Rev. Neurosci..

[122] Carmeliet P., Jain R.K. (2011). Principles and mechanisms of vessel normalization for cancer and other angiogenic diseases. Nat. Rev. Drug Discov..

[123] Zhuang P.Y., Wang J.D., Tang Z.H., Zhou X.P., Quan Z.W., Liu Y.B., Shen J. (2015). Higher proliferation of peritumoral endothelial cells to IL-6/sIL-6R than tumoral endothelial cells in hepatocellular carcinoma. BMC Cancer.

[124] Dunn G.P., Rinne M.L., Wykosky J., Genovese G., Quayle S.N., Dunn I.F., Agarwalla P.K., Chheda M.G., Campos B., Wang A. (2012). Emerging insights into the molecular and cellular basis of glioblastoma. Genes Dev..

[125] Fernandes C., Costa A., Osorio L., Lago R.C., Linhares P., Carvalho B., Caeiro C., De Vleeschouwer S. (2017). Current Standards of Care in Glioblastoma Therapy. Glioblastoma.

[126] Bulstrode H., Johnstone E., Marques-Torrejon M.A., Ferguson K.M., Bressan R.B., Blin C., Grant V., Gogolok S., Gangoso E., Gagrica S. (2017). Elevated FOXG1 and SOX2 in glioblastoma enforces neural stem cell identity through transcriptional control of cell cycle and epigenetic regulators. Genes Dev..

[127] Caren H., Stricker S.H., Bulstrode H., Gagrica S., Johnstone E., Bartlett T.E., Feber A., Wilson G., Teschendorff A.E., Bertone P. (2015). Glioblastoma Stem Cells Respond to Differentiation Cues but Fail to Undergo Commitment and Terminal Cell-Cycle Arrest. Stem Cell Rep..

[128] Lee J., Kotliarova S., Kotliarov Y., Li A., Su Q., Donin N.M., Pastorino S., Purow B.W., Christopher N., Zhang W. (2006). Tumor stem cells derived from glioblastomas cultured in bFGF and EGF more closely mirror the phenotype and genotype of primary tumors than do serum-cultured cell lines. Cancer Cell.

[129] Singh S.K., Hawkins C., Clarke I.D., Squire J.A., Bayani J., Hide T., Henkelman R.M., Cusimano M.D., Dirks P.B. (2004). Identification of human brain tumour initiating cells. Nature.

[130] Jiang Y., Uhrbom L. (2012). On the origin of glioma. Upsala J. Med. Sci..

[131] Sundar S.J., Hsieh J.K., Manjila S., Lathia J.D., Sloan A. (2014). The role of cancer stem cells in glioblastoma. Neurosurg. Focus.

[132] Ignatova T.N., Kukekov V.G., Laywell E.D., Suslov O.N., Vrionis F.D., Steindler D.A. (2002). Human cortical glial tumors contain neural stem-like cells expressing astroglial and neuronal markers in vitro. Glia.

[133] Galli R., Binda E., Orfanelli U., Cipelletti B., Gritti A., De Vitis S., Fiocco R., Foroni C., Dimeco F., Vescovi A. (2004). Isolation and characterization of tumorigenic, stem-like neural precursors from human glioblastoma. Cancer Res..

[134] Yuan X., Curtin J., Xiong Y., Liu G., Waschsmann-Hogiu S., Farkas D.L., Black K.L., Yu J.S. (2004). Isolation of cancer stem cells from adult glioblastoma multiforme. Oncogene.

[135] Clark P.A., Iida M., Treisman D.M., Kalluri H., Ezhilan S., Zorniak M., Wheeler D.L., Kuo J.S. (2012). Activation of multiple ERBB family receptors mediates glioblastoma cancer stem-like cell resistance to EGFR-targeted inhibition. Neoplasia.

[136] Bao S., Wu Q., McLendon R.E., Hao Y., Shi Q., Hjelmeland A.B., Dewhirst M.W., Bigner D.D., Rich J.N. (2006). Glioma stem cells promote radioresistance by preferential activation of the DNA damage response. Nature.

[137] Cruceru M.L., Neagu M., Demoulin J.B., Constantinescu S.N. (2013). Therapy targets in glioblastoma and cancer stem cells: Lessons from haematopoietic neoplasms. J. Cell. Mol. Med..

[138] Huang Z., Cheng L., Guryanova O.A., Wu Q., Bao S. (2010). Cancer stem cells in glioblastoma--molecular signaling and therapeutic targeting. Protein Cell.

[139] Eramo A., Ricci-Vitiani L., Zeuner A., Pallini R., Lotti F., Sette G., Pilozzi E., Larocca L.M., Peschle C., De Maria R. (2006). Chemotherapy resistance of glioblastoma stem cells. Cell Death Differ..

[140] Ikushima H., Todo T., Ino Y., Takahashi M., Saito N., Miyazawa K., Miyazono K. (2011). Glioma-initiating cells retain their tumorigenicity through integration of the Sox axis and Oct4 protein. J. Biol. Chem..

[141] Gangemi R.M., Griffero F., Marubbi D., Perera M., Capra M.C., Malatesta P., Ravetti G.L., Zona G.L., Daga A., Corte G. (2009). SOX2 silencing in glioblastoma tumor-initiating cells causes stop of proliferation and loss of tumorigenicity. Stem Cells.

[142] Song W.S., Yang Y.P., Huang C.S., Lu K.H., Liu W.H., Wu W.W., Lee Y.Y., Lo W.L., Lee S.D., Chen Y.W. (2016). Sox2, a stemness gene, regulates tumor-initiating and drug-resistant properties in CD133-positive glioblastoma stem cells. J. Chin. Med. Assoc..

[143] Su H.T., Weng C.C., Hsiao P.J., Chen L.H., Kuo T.L., Chen Y.W., Kuo K.K., Cheng K.H. (2013). Stem cell marker nestin is critical for TGF-beta1-mediated tumor progression in pancreatic cancer. Mol. Cancer Res..

[144] Gu G., Yuan J., Wills M., Kasper S. (2007). Prostate cancer cells with stem cell characteristics reconstitute the original human tumor in vivo. Cancer Res..

[145] Klein W.M., Wu B.P., Zhao S., Wu H., Klein-Szanto A.J., Tahan S.R. (2007). Increased expression of stem cell markers in malignant melanoma. Mod. Pathol..

[146] Singh S.K., Clarke I.D., Terasaki M., Bonn V.E., Hawkins C., Squire J., Dirks P.B. (2003). Identification of a cancer stem cell in human brain tumors. Cancer Res..

[147] Liu G., Yuan X., Zeng Z., Tunici P., Ng H., Abdulkadir I.R., Lu L., Irvin D., Black K.L., Yu J.S. (2006). Analysis of gene expression and chemoresistance of CD133+ cancer stem cells in glioblastoma. Mol. Cancer.

[148] Zhang M., Song T., Yang L., Chen R., Wu L., Yang Z., Fang J. (2008). Nestin and CD133: Valuable stem cell-specific markers for determining clinical outcome of glioma patients. J. Exp. Clin. Cancer Res..

[149] Lathia J.D., Gallagher J., Myers J.T., Li M., Vasanji A., McLendon R.E., Hjelmeland A.B., Huang A.Y., Rich J.N. (2011). Direct in vivo evidence for tumor propagation by glioblastoma cancer stem cells. PLoS ONE.

[150] Cheng L., Huang Z., Zhou W., Wu Q., Donnola S., Liu J.K., Fang X., Sloan A.E., Mao Y., Lathia J.D. (2013). Glioblastoma stem cells generate vascular pericytes to support vessel function and tumor growth. Cell.

[151] Chen H.Y., Lin L.T., Wang M.L., Laurent B., Hsu C.H., Pan C.M., Jiang W.R., Chen P.Y., Ma H.I., Chen Y.W. (2017). Musashi-1 Enhances Glioblastoma Cell Migration and Cytoskeletal Dynamics through Translational Inhibition of Tensin3. Sci. Rep..

[152] Glazer R.I., Vo D.T., Penalva L.O. (2012). Musashi1: An RBP with versatile functions in normal and cancer stem cells. Front. Biosci..

[153] Vaidya M., Bacchus M., Sugaya K. (2018). Differential sequences of exosomal NANOG DNA as a potential diagnostic cancer marker. PLoS ONE.

[154] Bien-Moller S., Balz E., Herzog S., Plantera L., Vogelgesang S., Weitmann K., Seifert C., Fink M.A., Marx S., Bialke A. (2018). Association of Glioblastoma Multiforme Stem Cell Characteristics, Differentiation, and Microglia Marker Genes with Patient Survival. Stem Cells Int..

[155] Seymour T., Nowak A., Kakulas F. (2015). Targeting Aggressive Cancer Stem Cells in Glioblastoma. Front. Oncol..

[156] Calabrese C., Poppleton H., Kocak M., Hogg T.L., Fuller C., Hamner B., Oh E.Y., Gaber M.W., Finklestein D., Allen M. (2007). A perivascular niche for brain tumor stem cells. Cancer Cell.

[157] Sanai N., Alvarez-Buylla A., Berger M.S. (2005). Neural stem cells and the origin of gliomas. N. Engl. J. Med..

[158] Seano G. (2018). Targeting the perivascular niche in brain tumors. Curr. Opin. Oncol..

[159] Bao S., Wu Q., Sathornsumetee S., Hao Y., Li Z., Hjelmeland A.B., Shi Q., McLendon R.E., Bigner D.D., Rich J.N. (2006). Stem cell-like glioma cells promote tumor angiogenesis through vascular endothelial growth factor. Cancer Res..

[160] Galan-Moya E.M., Le Guelte A., Lima Fernandes E., Thirant C., Dwyer J., Bidere N., Couraud P.O., Scott M.G., Junier M.P., Chneiweiss H. (2011). Secreted factors from brain endothelial cells maintain glioblastoma stem-like cell expansion through the mTOR pathway. EMBO Rep..

[161] Zhu T.S., Costello M.A., Talsma C.E., Flack C.G., Crowley J.G., Hamm L.L., He X., Hervey-Jumper S.L., Heth J.A., Muraszko K.M. (2011). Endothelial cells create a stem cell niche in glioblastoma by providing NOTCH ligands that nurture self-renewal of cancer stem-like cells. Cancer Res..

[162] Infanger D.W., Cho Y., Lopez B.S., Mohanan S., Liu S.C., Gursel D., Boockvar J.A., Fischbach C. (2013). Glioblastoma stem cells are regulated by interleukin-8 signaling in a tumoral perivascular niche. Cancer Res..

[163] Folkins C., Shaked Y., Man S., Tang T., Lee C.R., Zhu Z., Hoffman R.M., Kerbel R.S. (2009). Glioma tumor stem-like cells promote tumor angiogenesis and vasculogenesis via vascular endothelial growth factor and stromal-derived factor 1. Cancer Res..

[164] Thirant C., Galan-Moya E.M., Dubois L.G., Pinte S., Chafey P., Broussard C., Varlet P., Devaux B., Soncin F., Gavard J. (2012). Differential proteomic analysis of human glioblastoma and neural stem cells reveals HDGF as a novel angiogenic secreted factor. Stem Cells.

[165] Guichet P.O., Guelfi S., Teigell M., Hoppe L., Bakalara N., Bauchet L., Duffau H., Lamszus K., Rothhut B., Hugnot J.P. (2015). Notch1 stimulation induces a vascularization switch with pericyte-like cell differentiation of glioblastoma stem cells. Stem Cells.

[166] Brat D.J., Castellano-Sanchez A.A., Hunter S.B., Pecot M., Cohen C., Hammond E.H., Devi S.N., Kaur B., Van Meir E.G. (2004). Pseudopalisades in glioblastoma are hypoxic, express extracellular matrix proteases, and are formed by an actively migrating cell population. Cancer Res..

[167] Soeda A., Park M., Lee D., Mintz A., Androutsellis-Theotokis A., McKay R.D., Engh J., Iwama T., Kunisada T., Kassam A.B. (2009). Hypoxia promotes expansion of the CD133-positive glioma stem cells through activation of HIF-1alpha. Oncogene.

[168] Piccirillo S.G., Combi R., Cajola L., Patrizi A., Redaelli S., Bentivegna A., Baronchelli S., Maira G., Pollo B., Mangiola A. (2009). Distinct pools of cancer stem-like cells coexist within human glioblastomas and display different tumorigenicity and independent genomic evolution. Oncogene.

[169] Piccirillo S.G., Dietz S., Madhu B., Griffiths J., Price S.J., Collins V.P., Watts C. (2012). Fluorescence-guided surgical sampling of glioblastoma identifies phenotypically distinct tumour-initiating cell populations in the tumour mass and margin. Br. J. Cancer.

[170] Ruiz-Ontanon P., Orgaz J.L., Aldaz B., Elosegui-Artola A., Martino J., Berciano M.T., Montero J.A., Grande L., Nogueira L., Diaz-Moralli S. (2013). Cellular plasticity confers migratory and invasive advantages to a population of glioblastoma-initiating cells that infiltrate peritumoral tissue. Stem Cells.

